# Different Expressions of Pericardial Fluid MicroRNAs in Patients With Arrhythmogenic Right Ventricular Cardiomyopathy and Ischemic Heart Disease Undergoing Ventricular Tachycardia Ablation

**DOI:** 10.3389/fcvm.2021.647812

**Published:** 2021-03-19

**Authors:** Aleksandr A. Khudiakov, Daniil D. Panshin, Yulia V. Fomicheva, Anastasia A. Knyazeva, Ksenia A. Simonova, Dmitry S. Lebedev, Evgeny N. Mikhaylov, Anna A. Kostareva

**Affiliations:** ^1^Institute of Molecular Biology and Genetics, Almazov National Medical Research Centre, Saint Petersburg, Russia; ^2^Department of Bioengineering Systems, Saint Petersburg Electrotechnical University “LETI”, Saint Petersburg, Russia; ^3^Department of Women's and Children's Health, Center for Molecular Medicine, Karolinska Institute, Stockholm, Sweden

**Keywords:** microRNA, small RNA sequencing, pericardial fluid, arrhythmogenic (right ventricular) cardiomyopathy, expression analyses

## Abstract

**Introduction:** Pericardial fluid is enriched with biologically active molecules of cardiovascular origin including microRNAs. Investigation of the disease-specific extracellular microRNAs could shed light on the molecular processes underlying disease development. Arrhythmogenic right ventricular cardiomyopathy (ARVC) is an inherited heart disease characterized by life-threatening arrhythmias and progressive heart failure development. The current data about the association between microRNAs and ARVC development are limited.

**Methods and Results:** We performed small RNA sequence analysis of microRNAs of pericardial fluid samples obtained during transcutaneous epicardial access for ventricular tachycardia (VT) ablation of six patients with definite ARVC and three post-infarction VT patients. Disease-associated microRNAs of pericardial fluid were identified. Five microRNAs (hsa-miR-1-3p, hsa-miR-21-5p, hsa-miR-122-5p, hsa-miR-206, and hsa-miR-3679-5p) were found to be differentially expressed between patients with ARVC and patients with post-infarction VT. Enrichment analysis of differentially expressed microRNAs revealed their close linkage to cardiac diseases.

**Conclusion:** Our data extend the knowledge of pericardial fluid microRNA composition and highlight five pericardial fluid microRNAs potentially linked to ARVC pathogenesis. Further studies are required to confirm the use of pericardial fluid RNA sequencing in differential diagnosis of ARVC.

## Introduction

Pericardial fluid is a plasma ultrafiltrate found between the visceral and parietal pericardium, a double-walled sac surrounding the heart and roots of great vessels (1). Pericardial fluid is formed by the diffusion from both pericardial and epicardial vessels, as well as trans myocardial diffusion and acts as a lubricant, isolating, and protective layer for the beating heart (2). Due to a low clearance rate (3), pericardial fluid is enriched with heart-derived biologically active molecules, including cytokines, hormones, and nucleic acids (4–9). Thus, the composition of pericardial fluid potentially could influence the heart physiology and reflect certain heart conditions.

Among nucleic acids circulating in the pericardial fluid, extracellular microRNAs are of special interest. These short regulatory RNAs are extremely stable in the extracellular space due to the formation of RNA–protein complexes or packaging into vesicles, which protect them from nuclease digestion (10–14). Currently, data about pericardial microRNAs in patients with different structural heart diseases are lacking. A few studies reported on the differential expression of pericardial microRNAs in some common cardiovascular conditions. For instance, miR-423-5p was found to be enriched in pericardial fluid compared to serum; moreover, its levels differed in stable and unstable angina pectoris and aortic stenosis (15). Kuosmanen et al. (9) profiled microRNAs from the pericardial fluid of heart failure patients undergoing open-heart surgery and found no associations between microRNA profile and the clinical phenotype. In another study, exosomes from the pericardial fluid of patients were reported to contain myocardial-derived microRNAs promoting angiogenesis *in vitro* and *in vivo* (16). Taken together, these data suggest that microRNAs of pericardial fluid are biologically active molecules and could participate in cell-to-cell crosstalk.

Here, we focused on arrhythmogenic right ventricular cardiomyopathy (ARVC), an inherited disease difficult to diagnose and prognosticate (17, 18). At the early stages, ARVC is often asymptomatic or is manifested by heart rhythm abnormalities. During the ARVC progression, heart failure develops as a result of substitution of myocardium with fibrous and fat tissues (19). At the molecular level, ARVC is accompanied by significant changes in the signaling pathway activity (20–22). MicroRNAs as transcriptional regulators were reported to be involved in this pathological signaling network. Expression levels of miR-21 and miR-135b were found to be upregulated and downregulated correspondingly in the myocardium of ARVC patients (23). Transcriptome analysis of the HL-1 cells with PKP2 knockdown representing ARVC *in vitro* model revealed the downregulation of miR-184 (24). MicroRNA expression screening in plasma samples of ARVC patients and patients with idiopathic ventricular tachycardia (VT) revealed decreased expression of miR-320a associated with ARVC (25). Study performed on cardiac stromal cells disclosed three microRNAs (hsa-miR-520c-3p, hsa-miR-29b-3p, and hsa-miR-1183) differentially expressed between ARVC and control condition (26). Reduced blood plasma level of miR-320a-3p and elevated plasma levels of miR-144-3p, miR-145-5p, miR-185-5p, and miR-494-3p were reported to be associated with ARVC (27). A study involving transgenic mice carrying human DSG Q558* gene revealed different patterns of miRNA expression between the right and left ventricles: miR-217-5p and miR-708-5p were found to be upregulated and miR-499-5p was found to be downregulated specifically in the right ventricle (28). A recent study analyzing the microRNA expression profiles in blood and right ventricle tissue samples revealed that the expression of six microRNAs (miR-122-5p, miR-133a-3p, miR-133b, miR-142-3p, miR-182-5p, and miR-183-5p) was able to discriminate ARVC samples from healthy ones or other cardiomyopathy samples (29).

Although serum and myocardial microRNA expression evaluation has been reported, no common microRNA expression signature for ARVC is known. We suggested that microRNA composition of pericardial fluid in patients with ARVC might be specific, reflecting myocardial ARVC-related structural and molecular changes. We performed sequencing of microRNAs circulating in pericardial fluid of ARVC patients and patients with post-infarction VT (control group). We described the microRNA composition of pericardial fluid and performed an analysis of differentially expressed microRNAs.

## Materials and Methods

### Patient Characteristics

Patients referred for epicardial VT mapping and ablation between January 2019 and November 2020 were prospectively screened for inclusion into the study. Inclusion criteria were the following: indication to VT ablation; presumably epicardial VT exit site; a definite ARVC diagnosis, or the presence of a proven post-myocardial infarction scar; signed informed consent for the study. General exclusion criteria were the following: previous cardiothoracic surgery that potentially prevented pericardial manipulations; ongoing electrical storm (multiple defibrillation shocks in a short period of time); unsuccessful epicardial access; a previous ablation procedure <3 months ago; previous epicardial ablation; inadvertent right ventricle puncture during epicardial access, and/or visible blood in the pericardial fluid sample. Specific exclusion criteria for ARVC patients were other from “definite ARVC” diagnosis according to the established criteria; predominantly left ventricle disease and/or severe left ventricle systolic dysfunction (<40%); stenotic coronary artery disease; the history of angina and/or myocardial infarction. Specific exclusion criteria for ischemic group patients were severe right ventricle systolic dysfunction; indefinite myocardial scar; a coronary artery lesion requiring intervention. Patient clinical characteristics are summarized in Table 1.

**Table 1:** Patient demographics and clinical characteristics.

**Patient**	**Age**	**Sex**	**Disease-associated genetic variants**	**Diagnosis**	**Cardiac arrest**	**ICD implanted**	**LV EF, %**	**RV dysfunction**	**Heart failure, functional class**	Antiarrhythmic drugs^*^	**Number of VT induced**	**Previous ablation, >3 months**	**Previous ablation, <3 months**	**Acute ablation results**
1	73	M		CAD, post-MI	No	No	23%	No	II	BB	1	0	1	VT non-inducible
2	70	M		CAD, post-MI	No	Yes	40%	No	II	Amiodarone + BB	2	0	1	VT non-inducible
3	53	M		CAD, post-MI	No	Yes	30%	No	III	Sotalol	1	0	0	VT non-inducible
4	30	F	*PKP2* c.C235T, p.R79X, rs121434420	ARVC, definite	No	Yes	57%	No	I	BB	0	0	1	VT non-inducible
5	53	M	*PKP2* c.2509delA, p.S837Vfs, rs727504432, *DSG2* c.T3352A, p.S1118T	ARVC, definite	No	Yes	64%	Yes	I	Amiodarone + BB	1	0	0	VT non-inducible
6	59	F		ARVC, definite	No	Yes	67%	No	I	Amiodarone	0	0	0	VT non-inducible
7	38	M	*JUP* c.G2105A, p.R702H, rs200690479	ARVC, definite	No	Yes	60%	Yes	I	BB	0	0	0	VT non-inducible
8	20	M	*FLNC* c.G3800A, p.R1267Q, rs768767784	ARVC, definite	No	Yes	53%	No	I	BB	VF	3	1	Clinical VT non-inducible
9	24	M	*DSG2* c.G671A, p.S224N	ARVC, definite	No	No	50%	No	I	BB	0	0	0	VT non-inducible

*CAD, coronary artery disease; MI, myocardial infarction; ARVC, arrhythmogenic right ventricular cardiomyopathy; ICD, implantable cardioverter-defibrillator; VT, ventricular tachycardia; BB, beta-blocker; VF, ventricular fibrillation*.

* *Antiarrhythmic drugs present during the ablation procedure*.

### Pericardial Fluid Collection

Epicardial access was performed under general anesthesia *via* a subxiphoid transcutaneous puncture under fluoroscopic guidance, as described in detail earlier (30). Special attention was paid to enter the pericardial space without any damage to the right ventricle and to a minimal use of contrast media. Once a sheath was introduced into the pericardial space, pericardial fluid was aspirated into an empty sterile syringe. The fluid was visually assessed for the presence of blood; blood-contaminated samples were discarded. A blood sample was collected from a femoral vein sheath immediately after the pericardial access. Collected pericardial fluid was centrifuged at 3,000 g at 4°C for 15 min, and then supernatant was collected, aliquoted, and stored at −80°C.

### Genetic Testing

Genomic DNA was extracted from blood using FlexiGene DNA Kit (Qiagen). Target sequencing of 108 cardiomyopathy- and arrhythmia-associated genes was performed using Haloplex target enrichment (Agilent) with subsequent sequencing on MiSeq instrument (Illumina) as previously described (31). If disease-related genetic variants had not been identified, exome sequencing was performed as previously described (32). For the genetic variant verification, Sanger sequencing using a BigDye Terminator v3.1 kit and a 3,500 Genetic Analyzer (Applied Biosystems) was performed.

### RNA Extraction

Before RNA extraction, samples were additionally centrifuged at 3,000 g at 4°C for 15 min and the obtained supernatant was used for RNA extraction. Small RNAs were extracted using SPLIT RNA Extraction Kit (Lexogen) according to manufacturer's recommendations.

### Small RNA Library Preparation and Sequencing

Small RNA libraries were generated using Small RNA-Seq Kit (Lexogen) according to manufacturer's recommendations. The number of amplification cycles was 20 for all samples. Libraries were quantified using capillary gel electrophoresis using Bioanalyzer 2,100 (Agilent), then pooled in equimolar ratios based on 143-bp peak area, and purified in 6% PAAG gel using Gel Extraction Module (Lexogen). Sequencing was performed using MiSeq Reagent Kit v3 2x75bp and MiSeq equipment (Illumina) according to manufacturer's recommendations.

### Bioinformatic Analyses

Obtained paired-end reads were merged using FLASH tool (33), length filtered 15–31 bp using Geneious Prime 2020.0.5 (https://www.geneious.com), and then aligned to the mature microRNA database (miRbase, http://www.mirbase.org/) using Novoalign implemented in mirPRo tool (34). Counts were normalized, and differential expression was calculated using R Studio version 1.2.5019 (35) with R version 3.0.1 (36) DESeq2 package (37). Hierarchical clustering and data visualization were performed in Phantasus version 1.5.1 (https://artyomovlab.wustl.edu/phantasus/). Tissue-specific expression profile of microRNAs was determined using human microRNA tissue atlas (38). MicroRNA set enrichment analyses were performed using TAM 2.0 tool (http://www.lirmed.com/tam2/) (39). The data discussed in this publication have been deposited in NCBI's Gene Expression Omnibus (40) and are accessible through GEO Series accession number GSE164490.

### Quantitative PCR

Levels of selected microRNA were evaluated by qPCR. To remove heparin traces, RNA was treated with heparinase (Sigma) as was described before (41). For reverse transcription and qPCR microRNA-specific TaqMan Assays, TaqMan MicroRNA Reverse Transcription Kit and TaqMan Universal Master Mix II no UNG (all Thermo Fisher Scientific) were used according to manufacturer's recommendations. For miR-3679-5p measurement, a reverse transcription stem-loop primer and a primer pair for amplification were designed using sRNAPrimerDB online service (42). In this case, reverse transcription was performed using TaqMan MicroRNA Reverse Transcription Kit (Thermo Fisher Scientific), and real-time PCR was performed using qPCRmix-HS SYBR (Evrogen). TaqMan Assays and designed primer sequences are indicated in Supplementary Table 1.

### Correlation Analysis

Pearson's correlation coefficient (r) and *p*-value were calculated using GraphPad Prism v.5.00 to explore the association between small RNA sequencing data and real-time PCR data. Linear regression was used to plot the line of best fit shown in each graph.

## Results

### Patient Characteristics

Among 40 patients undergoing epicardial ablation during the study period, nine subjects were eligible according to the inclusion and exclusion criteria: six patients with definite ARVC according to 2010 ARVC Task Force Criteria (19) and three control patients with coronary artery disease and the history of previous myocardial infarction, with no suspicion toward inherited channelopathy syndromes (Table 1). Four out of six ARVC patients (patients 4, 5, 7, 9) carried genetic variants in genes (*PKP2, DSG2*, and *JUP*) coding for desmosomal proteins—plakophilin-2, desmoglein-2, and junctional plakoglobin. One ARVC patient (patient 8) carried a genetic variant in *FLNC* gene coding for actin-binding filamin C protein. Target sequencing and subsequent whole-exome sequencing did not reveal disease-linked genetic variants in patient 6.

### Presence of MicroRNA in Pericardial Fluid

Sequencing of microRNA revealed its presence in all pericardial fluid samples. In each sample, 145–411 microRNAs were detected, with the average number of 269 microRNAs per sample. Here, 105 microRNAs were shared between all samples (Table 2).

**Table 2:** List of microRNAs shared between all samples.

hsa-let-7a-3p	hsa-miR-26a-5p	hsa-miR-99b-5p	hsa-miR-181a-5p	hsa-miR-320c
hsa-let-7a-5p	hsa-miR-26b-3p	hsa-miR-99a-3p	hsa-miR-181b-5p	hsa-miR-335-3p
hsa-let-7b-5p	hsa-miR-26b-5p	hsa-miR-99a-5p	hsa-miR-181c-5p	hsa-miR-335-5p
hsa-let-7c-5p	hsa-miR-27a-3p	hsa-miR-100-5p	hsa-miR-181d-5p	hsa-miR-345-5p
hsa-let-7d-3p	hsa-miR-27b-3p	hsa-miR-101-3p	hsa-miR-182-5p	hsa-miR-361-3p
hsa-let-7d-5p	hsa-miR-28-3p	hsa-miR-106b-3p	hsa-miR-183-5p	hsa-miR-378a-3p
hsa-let-7e-5p	hsa-miR-28-5p	hsa-miR-122-5p	hsa-miR-186-5p	hsa-miR-421
hsa-let-7f-5p	hsa-miR-29a-3p	hsa-miR-125a-5p	hsa-miR-191-5p	hsa-miR-423-5p
hsa-let-7g-5p	hsa-miR-29c-3p	hsa-miR-125b-5p	hsa-miR-192-5p	hsa-miR-451a
hsa-let-7i-5p	hsa-miR-30a-3p	hsa-miR-130a-3p	hsa-miR-193a-5p	hsa-miR-484
hsa-miR-10a-3p	hsa-miR-30a-5p	hsa-miR-140-3p	hsa-miR-195-5p	hsa-miR-486-5p
hsa-miR-10a-5p	hsa-miR-30b-5p	hsa-miR-141-3p	hsa-miR-199a-3p	hsa-miR-497-5p
hsa-miR-10b-5p	hsa-miR-30c-5p	hsa-miR-143-3p	hsa-miR-199b-3p	hsa-miR-532-5p
hsa-miR-15b-5p	hsa-miR-30d-5p	hsa-miR-146a-5p	hsa-miR-200a-3p	hsa-miR-574-5p
hsa-miR-16-5p	hsa-miR-30e-3p	hsa-miR-146b-5p	hsa-miR-200b-3p	hsa-miR-652-3p
hsa-miR-19b-3p	hsa-miR-30e-5p	hsa-miR-148a-3p	hsa-miR-203a-3p	hsa-miR-744-5p
hsa-miR-21-5p	hsa-miR-34a-5p	hsa-miR-148b-3p	hsa-miR-204-5p	hsa-miR-769-5p
hsa-miR-22-3p	hsa-miR-92a-3p	hsa-miR-151a-3p	hsa-miR-221-3p	hsa-miR-888-5p
hsa-miR-23b-3p	hsa-miR-93-5p	hsa-miR-151a-5p	hsa-miR-222-3p	hsa-miR-1180-3p
hsa-miR-24-3p	hsa-miR-95-3p	hsa-miR-152-3p	hsa-miR-320a-3p	hsa-miR-1246
hsa-miR-25-3p	hsa-miR-98-5p	hsa-miR-181a-3p	hsa-miR-320b	hsa-miR-4286

### Description of Pericardial MicroRNAs

We analyzed microRNAs found in pericardial fluid according to the following criteria: (1) affiliation to a particular microRNA family; (2) cell- and tissue-specific expression. We identified 19 microRNA families presented in pericardial fluid by two or more microRNAs (Table 3). The most abundant in pericardial fluid microRNA family was the ubiquitous let-7 family with 11 detected microRNAs. Since the pericardial fluid could be enriched by cardiac microRNAs, we evaluated the presence of known cardiomyocyte-specific microRNAs: hsa-miR-1-3p, -133a-3p, -208a-3p, -208b-3p, -486-5p, and -486-3p. Hsa-miR-486-5p was detected in all samples, hsa-miR-1-3p was presented in six samples, hsa-miR-133a-3p in four samples, hsa-miR-486-3p was detected only in two samples, and hsa-miR-208a-3p and hsa-miR-208b-3p were not detected. Besides cardiomyocytes, the heart also consists of fibroblasts and endothelial cells. Thus, we identified in all pericardial fluid samples the microRNAs from miR-29 and miR-30 families, also having a high expression level in fibroblasts. Moreover, hsa-miR-21-5p, known to be expressed in cardiac fibroblasts (43), was presented in all studied samples. Two microRNAs (hsa-miR-93-5p; hsa-miR-106b-3p) from endothelial-specific family miR-17 were also detected in all pericardial fluid samples. Blood cell-derived microRNAs are also likely to contribute to pericardial fluid microRNA profile. Erythrocyte-specific hsa-miR-144-3p and hsa-miR-451a were detected in five samples and in all samples correspondingly.

**Table 3:** MicroRNA families with two or more microRNAs detected in all pericardial fluid samples.

**MicroRNA family**	**Number of microRNAs**	**MicroRNAs**
let-7	11	hsa-let-7a-3p; hsa-let-7a-5p; hsa-let-7b-5p; hsa-let-7c-5p; hsa-let-7d-3p; hsa-let-7d-5p; hsa-let-7e-5p; hsa-let-7f-5p; hsa-let-7g-5p; hsa-let-7i-5p; hsa-miR-98-5p
miR-10	9	hsa-miR-10a-3p; hsa-miR-10a-5p; hsa-miR-10b-5p; hsa-miR-99a-3p; hsa-miR-99a-5p; hsa-miR-99b-5p; hsa-miR-100-5p; hsa-miR-125a-5p; hsa-miR-125b-5p
miR-30	7	hsa-miR-30a-3p; hsa-miR-30a-5p; hsa-miR-30b-5p; hsa-miR-30c-5p; hsa-miR-30d-5p; hsa-miR-30e-3p; hsa-miR-30e-5p
miR-181	5	hsa-miR-181a-3p; hsa-miR-181a-5p; hsa-miR-181b-5p; hsa-miR-181c-5p; hsa-miR-181d-5p
miR-28	4	hsa-miR-28-3p; hsa-miR-28-5p; hsa-miR-151a-3p; hsa-miR-151a-5p
miR-8	3	hsa-miR-141-3p; hsa-miR-200a-3p; hsa-miR-200b-3p
miR-15	3	hsa-miR-15b-5p; hsa-miR-16-5p; hsa-miR-195-5p
miR-26	3	hsa-miR-26a-5p; hsa-miR-26b-3p; hsa-miR-26b-5p
miR-148	3	hsa-miR-148a-3p; hsa-miR-148b-3p; hsa-miR-152-3p
miR-320	3	hsa-miR-320a-3p; hsa-miR-320b; hsa-miR-320c
miR-17	2	hsa-miR-93-5p; hsa-miR-106b-3p
miR-25	2	hsa-miR-25-3p; hsa-miR-92a-3p
miR-27	2	hsa-miR-27a-3p; hsa-miR-27b-3p
miR-29	2	hsa-miR-29a-3p; hsa-miR-29c-3p
miR-95	2	hsa-miR-95-3p; hsa-miR-421
miR-146	2	hsa-miR-146a-5p; hsa-miR-146b-5p
miR-199	2	hsa-miR-199a-3p; hsa-miR-199b-3p
miR-221	2	hsa-miR-221-3p; hsa-miR-222-3p
miR-335	2	hsa-miR-335-3p; hsa-miR-335-5p

### Clustering and Differential Expression Analyses

**Figure 1:**
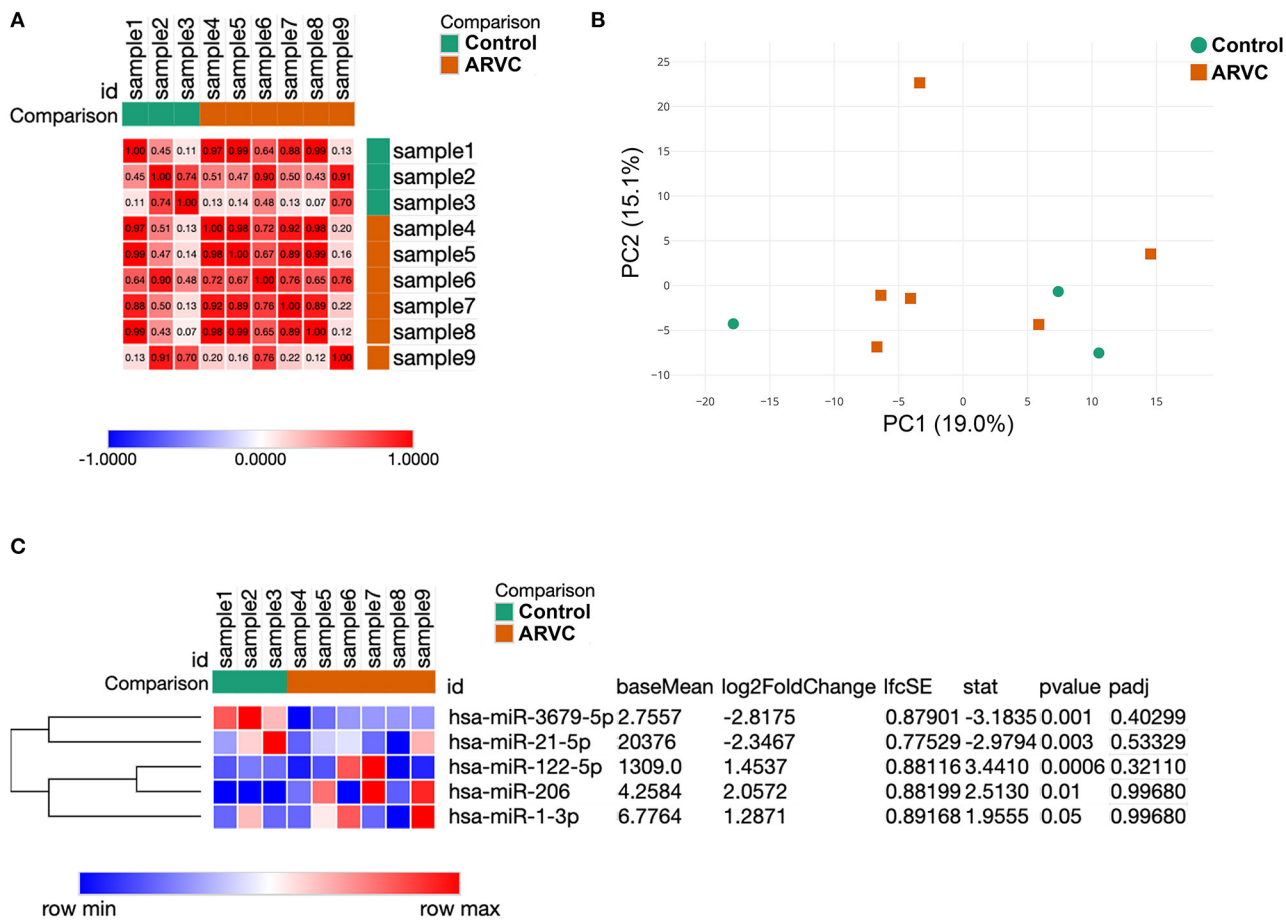
Results of samples clustering and microRNA differential expression analysis. **(A)** Similarity matrix of pericardial fluid samples calculated based on their microRNA expression patterns. **(B)** Principal component analysis of pericardial fluid samples. **(C)** Expression heatmap and result of statistical analyses of five differentially expressed microRNAs between control and arrhythmogenic right ventricular cardiomyopathy (ARVC) pericardial fluid samples.

Analysis of sample similarity revealed the high similarity of microRNA profiles of control group (post-infarction VT) and ARVC samples (Figure 1A). Principal component analysis (PCA) was performed in order to determine whether microRNA expression pattern is able to separate control and ARVC pericardial fluid samples. PCA showed no segregation of control or ARVC samples over the first two principal components (Figure 1B). Then, differential expression analysis was performed to reveal microRNA expression levels that significantly differ between patients with ARVC and the control group. Only microRNAs with at least one non-zero count between samples were taken into analyses. Although none of the microRNAs passed the multiple testing correction, we used non-corrected *p*-values taking into account the small group size and pilot nature of the study (Figure 1C). We found five differentially expressed microRNAs: two were downregulated in the ARVC group compared to the control group (hsa-miR-3679-5p and hsa-miR-21-5p), and three were upregulated in the ARVC group compared to the control group (hsa-miR-122-5p, hsa-miR-206, and hsa-miR-1-3p).

### Enrichment Analysis of Differentially Expressed MicroRNAs

**Figure 2:**
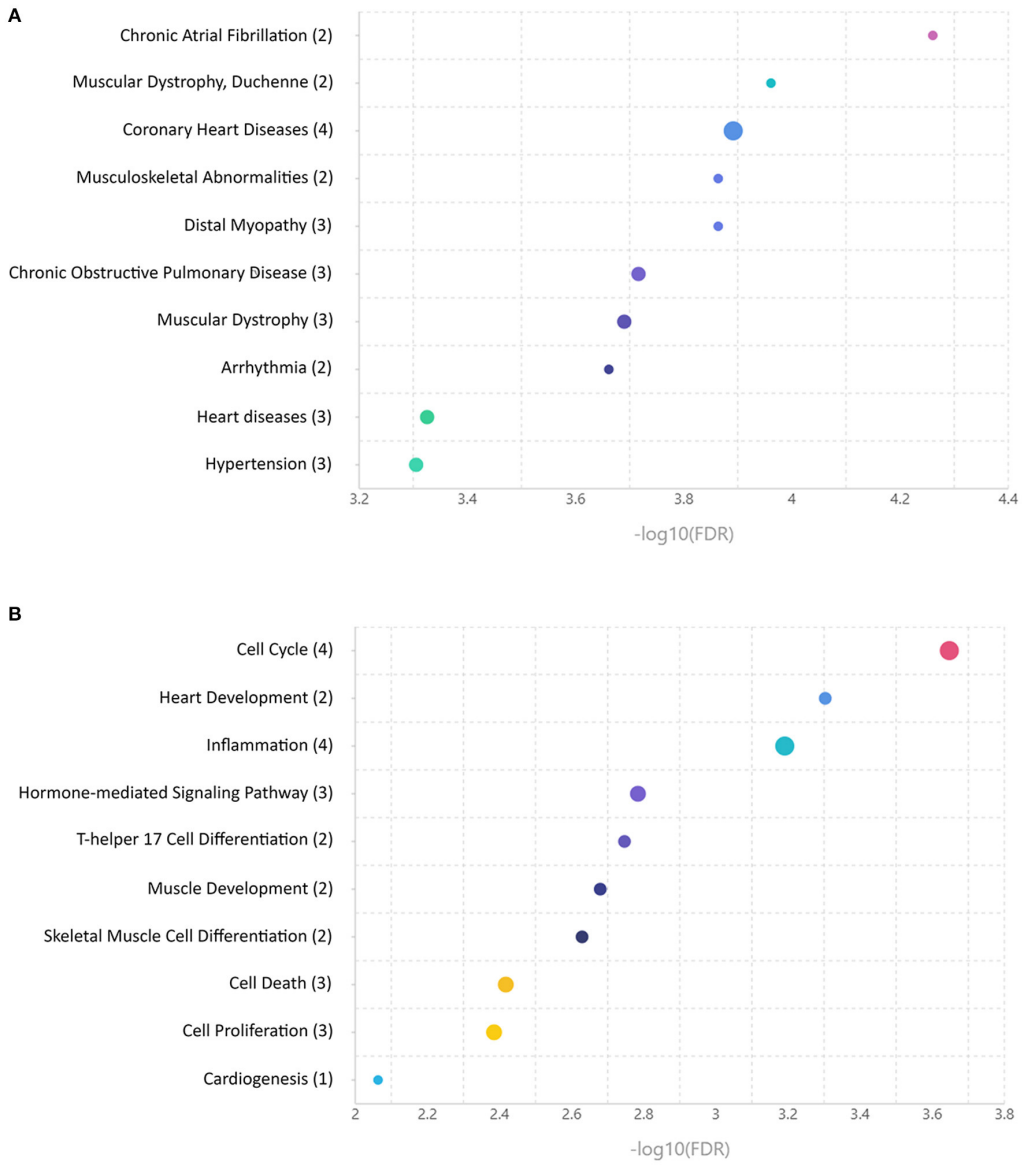
Enrichment analyses of microRNAs differentially expressed between control and arrhythmogenic right ventricular cardiomyopathy (ARVC) pericardial fluid samples. **(A)** MicroRNA set mapping against database containing disease-associated microRNA sets. **(B)** MicroRNA set mapping against database containing biological processes-associated microRNA sets. The number of microRNAs overlapping between the datasets is indicated in round brackets.

To provide the functional annotation of microRNAs differentially expressed between ARVC and control groups and to prove these microRNAs are associated with disease development, we performed microRNA set enrichment analysis. Comparison of differentially expressed microRNAs found in pericardial fluid with databases of disease-associated microRNAs showed overrepresentation of cardiovascular pathology terms—chronic atrial fibrillation, coronary heart disease, arrhythmia, heart diseases, and hypertension. Also, various muscular pathology terms were found including muscular dystrophy, musculoskeletal abnormalities, and distal myopathy (Figure 2A). MicroRNA set mapping against databases containing biological processes resulted in diverse terms including cell cycle, heart and muscle development, inflammation, hormone-mediated signaling pathway, T-helper 17 cell differentiation, muscle development, skeletal muscle cell differentiation, cell death, cell proliferation, and cardiogenesis (Figure 2B).

### Quantitative PCR Validation of Differentially Expressed MicroRNAs

**Figure 3:**
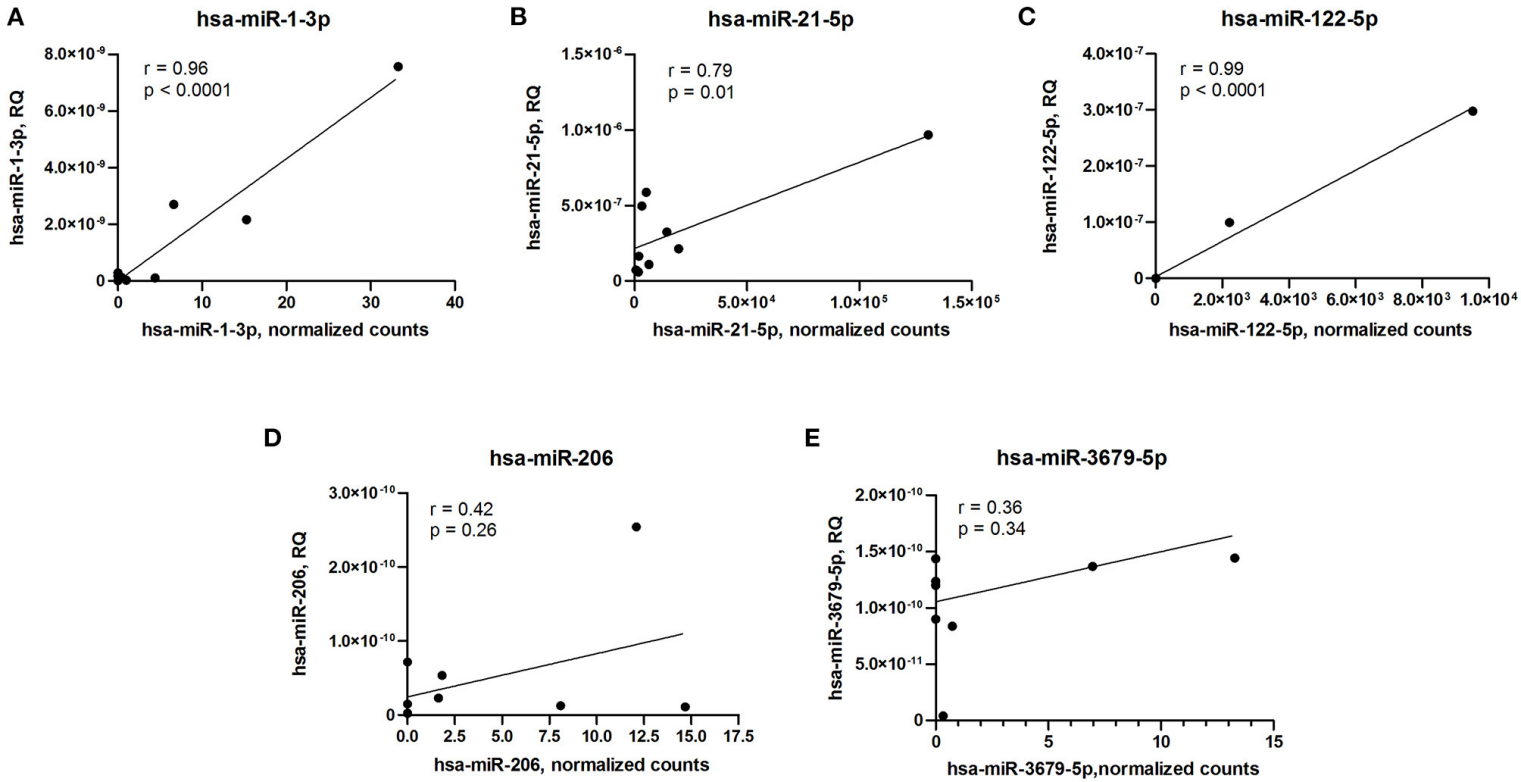
Correlation analyses of microRNA expression between qPCR and small RNA sequencing data. **(A)** hsa-miR-1-3p. **(B)** hsa-miR-21-5p. **(C)** hsa-miR-122-5p. **(D)** hsa-miR-206. **(E)** hsa-miR-3679-5p. RQ, relative quantity. Pearson's correlation coefficient (r) and *p*-value are indicated in each plot.

To validate microRNA expression data obtained by small RNA sequencing technique, we measured levels of differentially expressed microRNAs using qPCR and performed correlation analyses. Expression levels of three differentially expressed microRNAs (hsa-miR-1-3p, hsa-miR-21-5p, and hsa-miR-122-5p) measured by qPCR strongly correlated with sequencing data, whether expression levels of two other microRNAs (hsa-miR-206 and hsa-miR-3679-5p) demonstrated inconsistency between qPCR and small RNA sequencing (Figure 3).

## Discussion

In the present study, we performed profiling of microRNAs in pericardial fluid samples obtained from patients with ARVC or post-infarction VT patients. In contrast to previous studies that used open-heart surgery for pericardial fluid collection (9, 15, 16), we obtained pericardial fluid samples during transcutaneous epicardial access. This approach allowed us to obtain high-quality pericardial fluid samples from rare patient groups.

To date, qPCR is a gold standard approach to detect microRNAs including a circulating pool of microRNAs (44, 45). However, rapidly developing technologies of next-generation sequencing (NGS) allow to perform accurate quantitative and qualitative assessment of nucleic acids including small RNAs extracted from solid or liquid tissue samples (46–48). In the current study, we performed sequencing of microRNAs extracted from pericardial fluid samples, described the pericardial fluid microRNA composition, and performed the differential expression analysis with subsequent microRNA set enrichment analysis.

In line with previously published reports (9, 16), we detected microRNAs in all investigated samples and revealed a similar spectrum of pericardial fluid microRNAs, indicating the validity of NGS-based approaches for microRNA detection. Pericardial fluid is formed by the diffusion from both pericardial and epicardial vessels, as well as trans-myocardial diffusion. Consequently, pericardial fluid microRNA repertoire is at least partially formed by secretion of cardiac cells—cardiomyocytes, endothelial cells, and cardiac fibroblasts (9). Since most microRNAs are expressed in a broad spectrum of cell types and tissues, it is hardly possible to precisely determine their origin. However, we detected several microRNAs known to be expressed predominantly in fibroblasts (including cardiac fibroblasts), endothelial cells, or erythrocytes. As was previously reported (9, 15), cardiac-specific microRNAs were not present in all pericardial fluid samples and their average expression levels were low. This observation indicated the moderate release of these microRNAs from cardiomyocytes and likely the absence of acute myocardial injury in the studied patient group, as opposed to early stages of myocardial infarction, which is accompanied by the elevation of cardiac microRNA levels in serum (49).

It has been suggested that microRNA composition of pericardial fluid could reflect cellular and molecular events underlying cardiac pathologies (9). Consistent with a previous report (9), high levels of five microRNAs associated with cardiac disease (let-7b-5p, hsa-miR-16–5p, hsa-miR-21–5p, hsa-miR-125b-5p, and hsa-miR-451a) were found in pericardial fluid samples (Table 2). Some of these microRNAs could be potentially relevant for ARVC pathogenesis even in the absence of severe cardiac pathological remodeling.

Expression analysis revealed five microRNAs differentially expressed between ARVC and control groups. Among differentially expressed microRNAs, hsa-miR-1-3p and hsa-miR-21-5p were reported to be highly expressed by cardiomyocytes and cardiac fibroblast correspondingly (50, 51). These two microRNAs are well-known to contribute to various cardiovascular diseases including ischemic heart injury, atrial fibrillation, and cardiomyopathies of different origins (52). Hsa-miR-206 was reported to be highly expressed in skeletal muscle but can also be present in the myocardium. Heart-specific overexpression of hsa-miR-206 in transgenic mice led to Cx43 downregulation and subsequently contributed to abnormal heart rate and PR interval and shortened life span. At the same time, hsa-miR-122-5p and hsa-miR-3679-5p do not reveal any specific heart-expression profile; while the first one is present in the liver in high amounts (53) and in blood cells at lower levels (53), the second one does not have any tissue-specific expression pattern (38). Hsa-miR-122-5p is essential for embryonic liver development and also was reported to regulate multiple physiological and pathological processes in the adult liver (53, 54). Intriguingly, hsa-miR-122-5p levels in heart tissue and blood samples were shown to discriminate arrhythmogenic cardiomyopathy patients from unaffected family members and patients with other cardiomyopathies (29). In contrast, little is known about hsa-miR-3679-5p, the novel player in cardiovascular biology. Originally, it was discovered in peripheral blood (55). Later, two histone demethylases, KDM7A and KDM6A (UTX), were identified as hsa-miR-3679-5p direct targets in monocytes. Downregulation of these genes by hsa-miR-3679-5p led to the reduction of adhesion molecules and regulation of monocyte adhesion to endothelial cells, which could be linked to an inflammatory response (56). Future studies are needed to prove whether these processes are relevant to ARVC. Surprisingly, a link between differentially expressed microRNAs and fibrosis-related genes encoding for proteins responsible for extracellular matrix deposition was found. Four out of five differentially expressed microRNAs (hsa-miR-1-3p, hsa-miR-21-5p, hsa-miR-206, and hsa-miR-122-5p) were reported to regulate directly or indirectly matrix metalloproteinase 2 gene (43, 57–59) and genes coding for collagen isoforms (60–63). These microRNAs were also reported to regulate vimentin expression—a protein being a strong marker of mesenchymal cell- and fibroblast-specific intermediate filament (64–67).

In line with these data, the enrichment analysis found a strong association between differentially expressed microRNA set and cardiovascular diseases including persistent atrial fibrillation, coronary artery disease, unspecified heart disease, and arrhythmias. A number of associations with skeletal muscle pathologies were found likely due to the overlap of microRNA expression profiles between cardiac and skeletal muscle tissues. Analyses of associations with various biological processes found the associations with muscle development and differentiation and with basic biological processes like cell cycle, cell proliferation, and cell death. Moreover, an association with inflammation, which is frequently concomitant with the heart pathology, was observed.

Despite the fact that small RNA sequencing could be used for accurate microRNA quantification, the variation of detection levels between different methods and platforms was reported (68, 69). The combination of sequencing data with subsequent qPCR analyses of selected targets allows to take advantage of both techniques and validate the results using an independent approach (69). We performed correlation analyses of five differentially expressed microRNA levels measured by small RNA sequencing and qPCR. Surprisingly, only a partial correlation between small RNA sequencing and qPCR results was observed, and a similar fact was earlier reported in other studies (68, 69). In the present study, only three out of five microRNAs differentially expressed between ARVC and control group showed a strong correlation between the two techniques used for quantification.

Our study has several limitations. First, similarly to the previous studies, we were not able to profile pericardial fluid microRNAs of healthy subjects due to the invasive technique of sample collection. Consequently, as a result, there was an age difference between the experimental groups that could introduce additional bias to the microRNA expression. In addition, the study included a very limited number of patients meeting the inclusion criteria. At last, a low number of differentially expressed genes were identified, which restrict the power of enrichment analyses.

In conclusion, we performed microRNA profiling of pericardial fluid obtained from patients with recurrent VT due to ARVC or previous myocardial infarction using small RNA sequencing technique. We described the pericardial fluid microRNA composition and revealed five differentially expressed microRNAs. Once confirmed in future studies with a larger number of patients, these microRNAs might be used in differential diagnosis of structural heart diseases in patients undergoing invasive procedures involving epicardial access.

## Data Availability Statement

The datasets presented in this study can be found in online repositories. The names of the repository/repositories and accession number(s) can be found in the article/Supplementary Material.

## Ethics Statement

The studies involving human participants were reviewed and approved by Local ethical committee of Almazov National Medical Research Centre. The patients/participants provided their written informed consent to participate in this study.

## Author Contributions

AKh collected samples, performed experiments, analyzed the data, and wrote the paper. DP performed experiments and analyzed the data. YF collected samples and performed experiments. AKn collected samples and performed experiments. KS collected samples and co-wrote the paper. DL performed epicardial ablation, collected samples, and co-wrote the paper. EM performed epicardial ablation, collected samples, and co-wrote the paper. AKo supervised the research, acquired funding, and co-wrote the paper. All authors contributed to the article and approved the submitted version.

## Conflict of Interest

The authors declare that the research was conducted in the absence of any commercial or financial relationships that could be construed as a potential conflict of interest.
