# Supplementary material for: Different Expressions of Pericardial Fluid MicroRNAs in Patients With Arrhythmogenic Right Ventricular Cardiomyopathy and Ischemic Heart Disease Undergoing Ventricular Tachycardia Ablation
Source: Front Cardiovasc Med. 2021 Mar 19;8:647812. doi: 10.3389/fcvm.2021.647812 (PMC8017144; doi:10.3389/fcvm.2021.647812)
Supplement: Supplementary file 1 [file Table_1.DOCX]

**Supplementary table 1. TaqMan assays and primer sequences used for reverse transcription and qPCR.**

| **TaqMan assays (Thermo Fisher Scientific)** | |
| --- | --- |
| **Target** | **ID** |
| hsa-miR-1-3p | 002222 |
| hsa-miR-21-5p | 000397 |
| hsa-miR-122 | 002245 |
| hsa-miR-206 | 000510 |
| **Primer sequences (5’-3’)** | |
| hsa-miR-5679-5p reverse transcription | GTCGTATCCAGTGCAGGGTCCGAGGTATTCGCACTGGATACGACTCCCCT |
| hsa-miR-5679-5p qPCR forward | AACACGCTGAGGATATGGCA |
| hsa-miR-5679-5p qPCR reverse | GTCGTATCCAGTGCAGGGT |
